# Pilot-Scale Continuous Foam Fractionation for the Removal of Per- and Polyfluoroalkyl Substances (PFAS) from Landfill Leachate

**DOI:** 10.1021/acsestwater.2c00032

**Published:** 2022-05-04

**Authors:** Sanne J. Smith, Karin Wiberg, Philip McCleaf, Lutz Ahrens

**Affiliations:** † Department of Aquatic Sciences and Assessment, Swedish University of Agricultural Sciences (SLU), P.O. Box 7050, SE-750 07 Uppsala, Sweden; ‡ Uppsala Water and Waste AB, P.O. Box 1444, SE-751 44 Uppsala, Sweden

**Keywords:** per- and polyfluoroalkyl substances, water treatment, foam fractionation, landfill leachate, pilot-scale

## Abstract

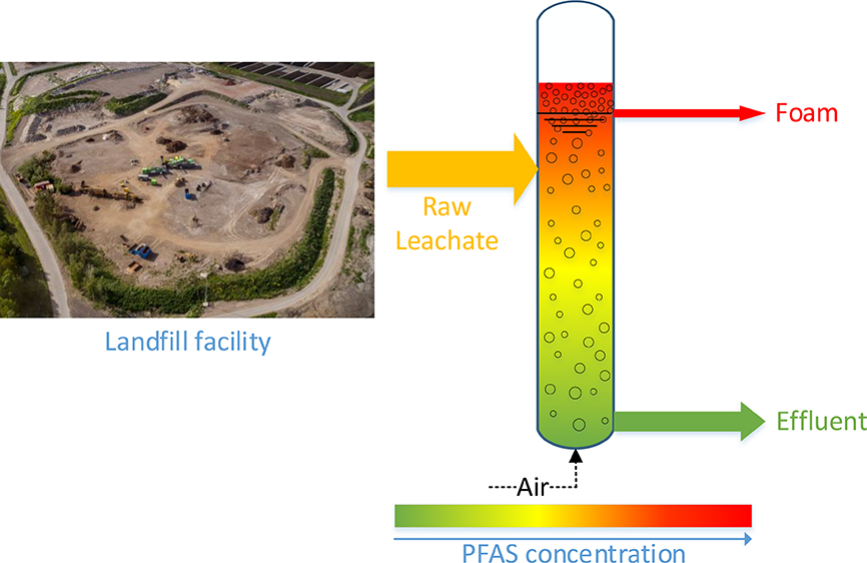

Per- and polyfluoroalkyl substances (PFAS) are of concern for their ubiquity in the environment combined with their persistent, bioaccumulative, and toxic properties. Landfill leachate is often contaminated with these chemicals, and therefore, the development of cost-efficient water treatment technologies is urgently needed. The present study investigated the applicability of a pilot-scale foam fractionation setup for the removal of PFAS from natural landfill leachate in a novel continuous operating mode. A benchmark batch test was also performed to compare treatment efficiency. The ΣPFAS removal efficiency plateaued around 60% and was shown to decrease for the investigated process variables air flow rate (*Q*_air_), collected foam fraction (%_foam_) and contact time in the column (*t*_c_). For individual long-chain PFAS, removal efficiencies above 90% were obtained, whereas the removal for certain short-chain PFAS was low (<30%). Differences in treatment efficiency between enriching mode versus stripping mode as well as between continuous versus batch mode were negligible. Taken together, these findings suggest that continuous foam fractionation is a highly applicable treatment technology for PFAS contaminated water. Coupling the proposed cost- and energy-efficient foam fractionation pretreatment to an energy-intensive degradative technology for the concentrated foam establishes a promising strategy for on-site PFAS remediation.

## Abstract 2

The process optimization of a continuous pilot-scale foam fractionation system resulted in promising PFAS removal efficiencies from landfill leachate.

## 1 Introduction

Per- and polyfluoroalkyl substances (PFAS) are a class of persistent, bioaccumulative, and toxic chemicals that have become widespread in the environment.^1^ They are used in consumer products, industrial applications, and firefighting foams for their high water and oil resistance, as well as for their surfactant properties.^2−4^ An increasing amount of research continues to show their extensive prevalence in the environment as well as their toxicity to both humans and animals.^5,6^ The most well-known class of PFAS are the perfluoroalkyl acids (PFAA), which encompass the perfluoroalkyl carboxylates (PFCA) and perfluoroalkanesulfonates (PFSA).^7^ These types of PFAS are commonly used as surfactants and can also be classified on the basis of the length of their hydrophobic perfluoroalkyl tail, with a total perfluorocarbon chain length below six for PFSA and seven for PFAA generally being considered short-chained (PFSA: C_*n*_F_2*n*+1_SO_3_H, *n* ≤ 5; PFCA: C_*n*_F_2*n*+1_COOH, *n* ≤ 6).^7,8^

Point sources of contaminated water are an important contributor to the origin of PFAS in the environment,^2^ implying that further pollution can be partially prevented by installing appropriate treatment technologies. Examples of such point sources include discharged leachate water from landfills, with total aqueous concentrations ranging from 100 to >100 000 ng L^–1^.^2,9,10^ PFAS in landfills originate from discarded consumer and industrial waste or PFAS-contaminated biosolids. Moreover, landfilled bottom ash from waste incinerators may still contain incompletely combusted PFAS. Biological leaching and physicochemical desorption of these PFAS result in their release to the landfill leachate, leading to high aqueous PFAS concentrations.^9,11^ Although the production and use of increasingly many PFAS are banned or restricted,^12,13^ landfills store previously produced waste over large timespans; hence, PFAS release from landfills is expected to remain a problem for the foreseeable future.^11^

With PFAS under widespread international scrutiny, limit values for discharge to the environment are becoming more stringent. In 2020, the European Food Safety Authority (EFSA) introduced a tolerably weekly intake of 4.4 ng of perfluorohexanesulfonic acid (PFHxS), perfluorooctanesulfonic acid (PFOS), perfluorooctanoic acid (PFOA), and perfluorononanoic acid (PFNA) per kilogram body weight per week.^14^ Consequently, to protect drinking water sources, many countries are starting to define concentration limits in environmental waters and hence enforcing treatment of contaminated effluents.^15−17^ Common wastewater treatment technologies, such as activated sludge or coagulation, are ineffective toward the removal of most PFAS.^18,19^ The current state of the art for PFAS removal from water is adsorption to granular activated carbon (GAC),^20^ but GAC needs to be regenerated often, is sensitive to matrix effects, and is less effective in the removal of short-chained PFAS.^21,22^ Hence, the development of alternative methods for the treatment of PFAS contaminated water is urgently needed.

Treatment methodologies can be broadly divided into removal and degradation techniques. Where removal technologies aim to concentrate PFAS into a waste fraction that is sent to further treatment, degradation technologies aim to mineralize the PFAS.^23^ Examples of removal methods include adsorption, membrane filtration, reverse osmosis, and ion exchange.^20,24−29^ Degradation methods include electrochemical oxidation, ultrasonication, advanced reduction processes, plasma treatment, and biological treatment.^20,24−31^ Degradation methods have the obvious advantage that the PFAS are destroyed rather than concentrated, but the formation of persistent transformation products can be an issue.^31^ Combining multiple removal and degradation approaches into a treatment train process is generally considered the most promising approach for future on-site PFAS remediation.^23^

A removal method that could be highly suitable as a first step in such a treatment train process is foam fractionation, which exploits the surfactant properties of common PFAS and has been applied successfully in full scale for the remediation of PFAS-contaminated groundwater.^32−34^ In foam fractionation, PFAS are adsorbed on the surface of gas bubbles rising through water. At the air–water interface, these bubbles form a foam that is enriched in PFAS, so separation and collapse of the foam results in a concentrated foamate and a relatively PFAS-free retentate.^35^ The process can be carried out in both batch and continuous operation. In continuous operation, stripping mode refers to operation with the liquid feed stream located above the liquid surface, whereas the feed enters below the foam/water interface in enriching mode.^35,36^

Foam fractionation is a suitable water treatment technology for dilute solutions using only air, thereby eliminating the need for chemicals, solvents, filter material, and adsorbents.^37^ Leachate water is a particularly complex matrix to treat, requiring extensive pretreatment before conventional PFAS treatment, such as GAC, ion exchange, or membrane filtration, can be applied successfully.^29^ These matrix effects are less problematic in the case of foam fractionation due to a beneficial effect of high ionic strength on the process performance and no risks of clogging or fouling of filter or membrane materials.^3,38,39^ Hence, foam fractionation has received increasing attention as a successful technology for PFAS removal from landfill leachate.^36,40,41^ However, its applicability is not limited to leachate water but extends to PFAS-contaminated groundwater, process water, and wastewater.^33,34,36,40,42^

An important limitation of foam fractionation is the low removal efficiency of short-chain PFAS.^3,33,38,40−42^ Metal cation activators can be used to increase the removal, but this effect has not been shown for short-chain substances.^38,40,43^ ΣPFAS removal has further been shown to increase for increasing aeration time,^3,38,40,42^ gas flow rate,^3,32,33,40^ and ionic strength^3,38,42^ and for decreasing initial PFAS concentration.^3,42,43^ However, for low initial PFAS concentrations (<50 ng L^–1^), removal was instead observed to increase at increasing concentration for a wide range of compounds.^40^ The effect of pH is ambiguous, with some studies reporting more efficient treatment at low pH,^43^ others at intermediate pH,^3^ and others at high pH.^32^ Most probably, this is because other operating conditions are more influential than pH. Finally, the PFAS concentration in the foam has been shown to depend on the collected foam volume.^3,41^

Reported removal efficiencies strongly depend on the types of PFAS and water matrices under investigation but generally range between 0 and <50% for short-chain PFCA,^33,34,38,40−42^ while for long-chain PFAS, efficiencies can reach up to >99%.^3,34,38,40−43^ Most work on PFAS removal with foam fractionation has been done in batch mode, with easy control of contact time and effluent quality.^3,32,40−43^ However, recent exploratory work by McCleaf et al.^40^ has indicated that similar removal efficiencies can be reached in continuous operation, which comes with operational advantages in larger scale applications, but until now no pilot-scale results have been presented in academic literature.

The present study aimed to assess the effect of operational parameters on PFAS removal from landfill leachate with continuous pilot-scale foam fractionation. The specific objectives were to (i) determine the effectiveness of this technology in a continuous setup, (ii) for the first time, systematically evaluate the effect of different operating parameters on this continuous pilot scale process, and (iii) test real landfill leachate on-site and thereby avoid effects of sedimentation and chemical or microbiological changes during transport. The findings advance the understanding of the opportunities provided by the use of foam fractionation for PFAS removal from contaminated water.

## 2 Materials and Methods

### 2.1 Treatment setup

**Figure 1:**
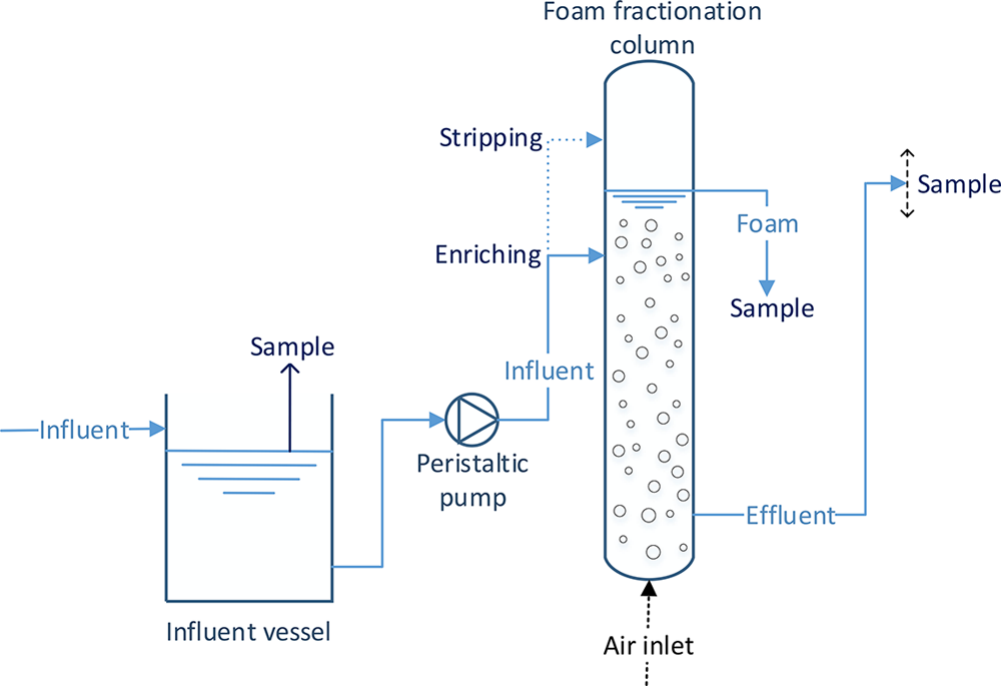
Process flow diagram of the continuous foam fractionation treatment. Column ø: 19 cm, water surface 1.63 m above column bottom. The height of the effluent hose was adaptable, which was used to control the foam and effluent flow rates. In enriching mode, the influent entered the column below the water surface (solid line). In stripping mode, the influent entered above the water surface (dotted line).

A 19 cm diameter polypropylene (PP) column was used for all experiments with the water surface at 1.63 m height above the column bottom. Leachate water from the Hovgården landfill in Uppsala, Sweden was collected in real time from the inflow to the on-site water treatment plant. The influent vessel (PP, 300 L) was mixed by the inflow of leachate. All leachate originated from the same pumping station, thus excluding leachate from an area of the landfill where sludge from a municipal wastewater treatment plant is stored. All tests were done on days with similar weather profiles to exclude effects due to fluctuations in water quality as much as possible. A peristaltic pump with variable flow rate (Watson Marlow, 630SN/RE with Pureweld Xl 12.7 mm tubing) supplied a steady leachate flow to the column. The leachate entered the column under the water surface in enriching mode, at a height of 1.43 m above the column bottom (Figure 1). In stripping mode, the influent entered above the water surface at a height of 1.83 m above the column bottom. All experiments were done at room temperature.

Air was dispersed at the bottom of the column using four brass diffusers, each with 18 mm diameter and 30 mm length, attached to a stainless steel manifold. The airflow was controlled with a rotameter (0–20 L min^–1^, ZYIA instrument company, FL3-1). The column top was sealed and nearly airtight so all inlet air exited the column at the foam exit surface, carrying with it foam accumulated at the water surface. The foam collection was optimized by changing the height of the effluent outlet, thereby controlling the effluent flow as well. The foam flow was measured at least every 30 min with a PP volumetric flask. A process overview of the treatment setup is given in Figure 1.

The independent variables in all experiments were contact time (*t*_c_, min), air flow (*Q*_air_, L min^–1^), and foam fraction (%_foam_, %). The *t*_c_ was assessed at both constant *Q*_air_ and at constant air-to-feed ratio (AR). The *t*_c_ and AR were not entirely independent, since both are functions of the water flow rate (*Q*_W_), as given in eqs 1 and 2, with *V*_column_ as the water volume in the column. The foam fraction was defined as in eq 3, with *Q*_F_ as the foam flow (L min^–1^).
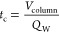
1
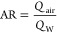
2
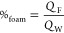
3

### 2.2 Experimental Approach

To confirm the independence of sampling time on the removal in continuous operation shown by McCleaf et al.,^40^ a 30 min continuous initial experiment was performed in triplicate at 10 min *t*_c_, 10 L min^–1^*Q*_air_, and 30% foam. In these tests, approximately 250 mL of influent from the influent vessel was collected in clean PP bottles initially, 150 mL of foam and 250 mL of effluent were sampled after both 15 and 30 min treatment time from their respective exit hoses (without the use of a vacuum pump), and 250 mL of water from directly under the air/water interface was sampled after 30 min with a vacuum pump (GAST, DOA-P704-AA) connected to a PVC hose that was inserted approximately 5 cm below the water surface.

In this initial experiment, no significant differences between the effluent at 15 min and the effluent at 30 min were found in the concentrations of individual compounds as well as groups (paired *t* test, all *p* > 0.05). Detailed results, including the difference between sampling the effluent from the bottom as compared to the top of the column, are given in the Supporting Information (SI) Section A (Figure S1). On the basis of this stability in effluent over time, all subsequent continuous experiments (Table 1, all except Exp. 0 and 15) were run once for a total duration of 2 h, with replicate influent, effluent, and foam samples taken at four different time points (30, 60, 90, and 120 min) instead of in experimental triplicates. Approximately 250 mL of influent and effluent and 150 mL of foam were collected in clean PP bottles at each sampling time point. Average influent, effluent, and foam concentrations were calculated from the four different samples per type for each experiment to assess the effects of *t*_c_, *Q*_air_ and %_foam_. A detailed overview of all experiments is given in Table 1, and the dates on which the experiments were performed are given in Table S1.

**Table 1:** Overview of All Experiments^a^

Exp.	contact time (min)	air flow (L min^–1^)	targeted foam fraction (%)	water flow rate in (L min^–1^)	foam flow rate (L min^–1^)	effluent flow rate (L min^–1^)	air ratio	operating mode
**0**	10	10	30	4.6	0.46	4.2	2.2	enriching
**1**	10	20	10	4.6	0.46	4.2	4.3	enriching
**2**	30	6.7	10	1.5	0.15	1.4	4.3	enriching
**3**	10	20	10	4.6	0.46	4.2	4.3	stripping
**4**	30	13	20	1.5	0.31	1.2	8.7	stripping
**5**	30	13	20	1.5	0.31	1.2	8.7	enriching
**6**	20	20	10	2.3	0.23	2.1	8.7	enriching
**7**	20	10	10	2.3	0.23	2.1	4.3	enriching
**8**	20	10	20	2.3	0.46	1.8	4.3	enriching
**9**	20	10	30	2.3	0.69	1.6	4.3	enriching
**10**	20	5.0	20	2.3	0.46	1.8	2.2	enriching
**11**	20	20	20	2.3	0.46	1.8	8.7	enriching
**12**	30	20	10	1.5	0.15	1.4	13	enriching
**13**	15	13	10	3.1	0.31	2.8	4.3	enriching
**14**	20	10	5	2.3	0.12	2.2	4.3	enriching
**15**	20	20	3	batch mode - not applicable				
**16**	15	20	10	3.1	0.31	2.8	6.5	enriching
**17**	20	7.5	20	2.3	0.46	1.8	3.2	enriching

a See Figure 1 for the difference between stripping and enriching modes.

Additionally, a set of triplicate batch experiments was carried out (Table 1, Exp. 15) to investigate the difference between continuous and batch operation. Here, the column was filled up to 1.57 m height and an air flow of 20 L/min was applied for 20 min contact time. During the first 15 min, foam collection was identical to the continuous tests, but during the final 5 min, foam was also collected with a vacuum pump to increase the collected foam fraction. Effluent samples were taken from sampling points on both the bottom and the top of the column, to compare the effect of sampling height.

### 2.3 PFAS Analysis

In total, 29 PFAS were included in analytical method, namely, 11 PFCA (PFBA, PFPeA, PFHxA, PFHpA, PFOA, PFNA, PFDA, PFUnDA, PFDoDA, PFTriDA, PFTeDA), 7 PFSA (PFBS, PFPeS, PFHxS, PFHpS, PFOS, PFNS, PFDS), 3 fluorotelomer sulfonates (4:2 FTSA, 6:2 FTSA, 8:2 FTSA), the two components of F-53B (9Cl-PF3ONS and 11Cl-PF3OUdS),^44^ HFPO-DA (trade name GenX), FOSA, MeFOSAA, EtFOSAA, NaDONA, and PFECHS. Twenty mass-labeled internal standards (IS) were used, which were spiked to the samples before extraction (Wellington Laboratories, MPFAC-24ES mixture with ^13^C_3_-HFPO-DA added individually): ^13^C_4_-PFBA, ^13^C_5_-PFPeA, ^13^C_5_-PFHxA, ^13^C_4_-PFHpA, ^13^C_8_-PFOA, ^13^C_9_-PFNA, ^13^C_6_-PFDA, ^13^C_7_-PFUnDA, ^13^C_3_-PFDoDA, ^13^C_2_-PFTeDA, ^13^C_3_-PFBS, ^13^C_3_-PFHxS, ^13^C_8_-PFOS, ^13^C_2_-4:2 FTSA, ^13^C_2_-6:2 FTSA, ^13^C_2_-8:2 FTSA, ^13^C_3_-HFPO-DA, ^13^C_8_-FOSA, D_3_-MeFOSAA, and D_5_-EtFOSAA (for full names and other details of the native PFAS and IS see Tables S3 and S4).

The collected samples were filtered through glass microfiber filters (47 mm diameter, Whatman, China), weighed, and subsequently analyzed for PFAS concentration using solid phase extraction (SPE) followed by ultraperformance liquid chromatography tandem mass-spectrometry (UPLC–MS/MS) analysis. The SPE method has been described previously^22,45^ (see also Section C in the Supporting Information).

A SCIEX Triple Quad 3500 UPLC–MS/MS system was used for PFAS analysis. Twenty microliters of extract was injected on a Phenomenex Gemini 1.7 μm C18 HPLC column with a Phenomenex KJ0-4282 analytical guard column and a Phenomenex Kinetix 1.7 μm C18 precolumn, all at 40 °C. A gradient of 0.6 mL/min 10 mM ammonium acetate in Milli-Q water and methanol was used for a total duration of 9 min per run. The initial gradient was set to 5% methanol, which was increased to 55% within the first 0.1 min. Then, it was further increased to 99% over 4.4 min, held there for 3.5 min, after which it was decreased again to 5% over 0.5 min and held there for another 0.5 min. The MS/MS was operated in scheduled multiple reaction monitoring (MRM) mode with negative electrospray ionization. For compounds with branched as well as linear isomers, only summed concentrations were reported. Details and quality control data on the analytical method are given in Tables S2–S4 in the SI Section C.

### 2.4 General Chemistry Analysis

For one continuous experiment and the batch experiment, 1 L influent, effluent, and foam samples were taken and shipped to ALS Scandinavian, Stockholm, Sweden for general chemistry analysis. For the preliminary triplicate continuous experiment and another continuous experiment, only influent and effluent were sampled and analyzed. The parameters were included in the analysis, and the results are given in Table S5.

### 2.5 Data Treatment

For each continuous test, mean concentrations of the four collected influent, effluent, and foam samples were calculated. The removal efficiency (RE) was calculated as in eq 4, with the standard deviation (σ_RE_) calculated as in eq 5. Here, *C*_EF_, *C*_IN_, σ_EF_, and σ_IN_ refer to the effluent and influent mean PFAS concentrations and corresponding standard deviations, respectively. The removal efficiency as a function of the independent variables (*x*) was fitted to eq 6 using the unweighted *fit* function in Matlab (version R2017B), with *k* and RE_Max_ as dependent empirical variables. This equation was selected because it converges to a horizontal asymptote and proved suitable for fitting the data, but other equations may be appropriate as well.
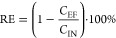
4
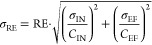
5
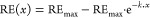
6

For PFCA and PFSA, the mean RE as a function of perfluoroalkyl chain length (*N*_c_) was fitted to eq 7, with *a* as the dependent empirical variable. Furthermore, a mass balance (MB) and its corresponding standard deviation (σ_MB_) were calculated for each experiment as per eqs 8 and 9, respectively, with *C*_Foam_ the mean concentration in the foam and σ_Foam_ the corresponding standard deviation. All statistical analyses, curve fitting, and plotting were done in Matlab, version R2017b.
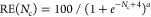
7
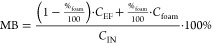
8
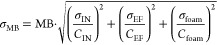
9

Values below the limit of quantification were taken as zero, which is acknowledged to introduce an error. However, substituting a fraction of the detection limit is known to introduce an equal level of inaccuracy.^46^ In all analyzed samples, the highest possible concentration of nondetect PFAS would contribute at most 0.4% to the ΣPFAS concentration (see SI Section C for details). This fraction was deemed negligible; hence, nondetect concentrations were set to zero. Some samples were contaminated or lost during the analysis, in which case the results were based on the remaining three samples. An overview of all tests for which samples were excluded is given in Table S6.

## 3 Results and Discussion

### 3.1 Leachate Characteristics

The average ΣPFAS concentration in the influent leachate was 2400 ± 400 ng L^–1^. Because the untreated leachate was collected from the influent of an operating treatment plant and the tests were carried out over different days, this level of variability falls within the expectations. The influent ΣPFAS consisted of 46 ± 10% short-chain PFCA, 27 ± 6% long-chain PFCA, 7 ± 1% short-chain PFSA, 15 ± 3% long-chain PFSA, and 4 ± 1% other types of PFAS (for details on the PFAS classification see Table S7). The influent ΣPFAS concentration was not found to affect the removal efficiency (Pearson’s *r* = −0.23 (95% CI: −0.71–0.40), *p* > 0.05). Of all PFAS included in the analysis, a statistically significant correlation between RE and influent concentration was only found for PFECHS, with the RE increasing at higher influent concentrations (Pearson’s *r* = 0.74 (95% CI: 0.28–0.92), *p* < 0.05). All PFAS included in the method were detected in at least one of the samples.

For leachate samples taken on testing dates, average influent dissolved organic carbon (DOC), conductivity, ammonium, and bicarbonate alkalinity were 36 mg L^–1^, 440 mS m^–1^, 59 mg L^–1^, and 1300 mg L^–1^, respectively. A selective overview of the mean general chemistry characteristics of the influent, effluent, and foam is given in Table 2, with the complete data set given in Table S5. DOC, iron, and aluminum were enriched in the foam, but otherwise no effects of the treatment on the general chemistry were found. Samples were not taken for each test, but the leachate composition from this pumping station at Hovgården is known to be very stable in terms of general chemistry characteristics. On the basis of 15 regularly distributed measurements in 2021, relative variations of the mean iron concentration (5.7 mg L^–1^), conductivity (510 mS m^–1^), pH (7.6), and total organic carbon (43 mg L^–1^) were only 15, 11, 3, and 10%, respectively.

**Table 2:** Overview of General Chemistry Data^a^

	influent (*n* = 4)	effluent (*n* = 4)	foam (*n* = 2)
DOC (mg L^–1^)	36	36	45
phosphor (μg L^–1^)	140	120	190
calcium (mg L^–1^)	150	150	150
manganese (μgL^-1^)	520	530	610
sodium (mg L^–1^)	710	710	740
potassium (mg L^–1^)	240	240	260
iron (mg L^–1^)	5.3	4.8	9.7
aluminum (μg L^–1^)	27	25	44
copper (μg L^–1^)	54	28	77
magnesium (mg L^–1^)	56	57	60
COD-Mn (mg L^–1^)	27	29	31
ammonium (mg L^–1^)	59	60	61
nitrate (mg L^–1^)	18	18	17
chloride (mg L^–1^)	920	910	950
sulfate (mg L^–1^)	130	120	95
conductivity (mS m^–1^)	440	450	440
pH	7.9	8.0	8.0
alkalinity (mg L^–1^)	1300	1300	1400
TOC (mg L^–1^)	36	34	48

a For the complete dataset, see Table S5.

### 3.2 Effect of Process Variables

**Figure 2:**
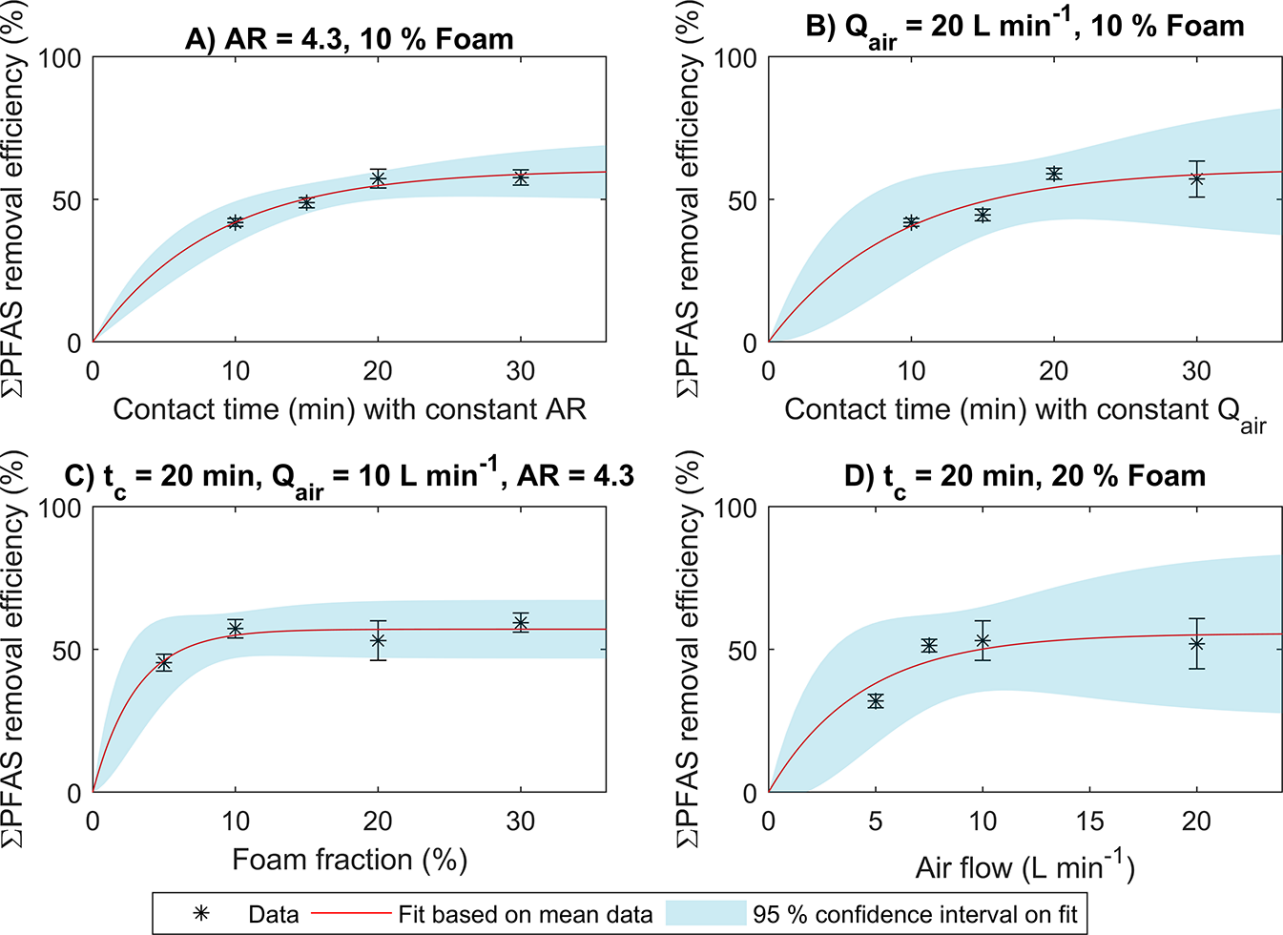
Effect of (A) contact time (*t*_c_) at a constant air ratio (AR) of 4.3, 10% foam; (B) *t*_c_ at a constant air flow (*Q*_air_) of 20 L min^–1^, 10% foam; (C) foam fraction (%_foam_) at constant *t*_c_ = 20 min, *Q*_air_ = 10 L min^–1^; AR = 4.3 and (D) *Q*_air_ at constant *t*_c_ = 20 min, 20% foam on the total PFAS removal. The red lines and blue shading represent a least-squares fit of the mean data to eq 6 with the corresponding 95% confidence interval of the fit, respectively. The experiments included in each plot in order of increasing *x*-value were (A) 1, 13, 7, and 2; (B) 1, 16, 6, and 12; (C) 14, 7, 8, and 9; and (D) 10, 17, 8, and 11 (Table 1).

The effect of all investigated process variables on the ΣPFAS removal is shown in Figure 2. Both at constant air-to-feed ratio (AR) and at constant air flow (*Q*_air_), decreasing the contact time (*t*_c_) below 20 min was shown to decrease the ΣPFAS removal efficiency (RE). Importantly, it was found that *t*_c_ also limits the removal while the AR is kept constant, although the effect may be different at higher AR values. This result indicates that increasing the *Q*_air_ cannot make up for a too short *t*_c_. These results are in good agreement with the results of Meng et al.,^3^ who found total aeration time to be one of the most influential variables in the performance of foam fractionation for PFAS removal from aqueous firefighting foam concentrate.

Altogether, the results strongly indicate that RE is negatively impacted by *t*_c_ values below 15 min, but the extent of decrease in RE is uncertain. The initial experiment at 10 min *t*_c_ (Exp. 0, Table 1, SI Section A) showed a higher RE of 47 ± 3% as compared to the RE found in Exp. 1 of 42 ± 1%, which indicates that higher ΣPFAS removal efficiencies may be achievable at a short *t*_c_ than is now shown in Figure 2A, B. Nonetheless, for the experiment in stripping mode at 10 min *t*_c_ (Exp. 3, Table 1), an even lower RE of 29 ± 4.7% was observed (Section 3.4), confirming the limited RE at low *t*_c_ values.

Collecting lower foam fractions lead to higher foam concentrations, as found from one-way ANOVA over the ΣPFAS concentration of all collected foam samples divided into groups based on their %_foam_ (F(4, 61) = 3.8, *p* < 0.05), which has also been found previously.^3,41^ Differences in foam concentration were only statistically significant between 30% foam as compared to 3% and 5% (*p* < 0.05) but statistically insignificant between the other groups. Decreasing the %_foam_ only affected the removal at fractions below 10%, which corresponds to Robey et al.’s^41^ finding that most of the removal occurs in the first 14% of volume removed. This is beneficial from a process design perspective, since achieving the same removal at a low %_foam_ leads to a lower volume of concentrated foam that needs secondary treatment. Since the %_foam_ is controlled by changing the effluent flow and the foam outlet is directly above the water–air surface, the collected foam was relatively wet. Strictly speaking, this mode of operation is a mix of bubble fractionation and foam fractionation, as explained by Lemlich.^35^ However, since foaming was observed in all tests, foam fractionation was chosen as terminology.

*Q*_air_ was shown to limit the removal at values below 7.5 L min^–1^ (Figure 2D). Since the removal is highly dependent on the surface area available for sorption, air flow is considered a very influential process variable.^35^ The air–liquid surface area further relates to the size of the introduced air bubbles.^35,42^ In the current study, the diffusers used generated relatively large air bubbles (up to approximately 5 mm diameter). Instead, the use of a membrane, glass frit, electrochemical bubble generation, or other technologies may increase the available surface area and thereby improve the removal.^40,42^

For all process parameters, their effect on the ΣPFAS RE fits well with the empirical model given by eq 6. For each run, the ΣPFAS removal was shown to plateau around 60%, with fitted RE_max_ values ranging between 56% and 61% for the effect of *Q*_air_ (Figure 2D) and the effect of *t*_c_ at constant *Q*_air_ (Figure 2B), respectively. The ΣPFAS RE is thus affected by all these variables, but the effect is limited and RE_Max_ does not reach 100%. Instead, it is also limited by the PFAS composition of the inlet water. The leachate water used in this study contained on average 46% short-chain PFCA, which were only marginally removed in the foam fractionation process. Therefore, the ΣPFAS removal reached a plateau at approximately 60%. It should be realized that the fitted RE_Max_ and *k* parameters obtained for each variable, given in Table S8, probably depend strongly on the inlet water composition and parameters such as PFAS composition and DOC concentration.

### 3.3 Effect of PFAS Composition and Chain Length

**Figure 3:**
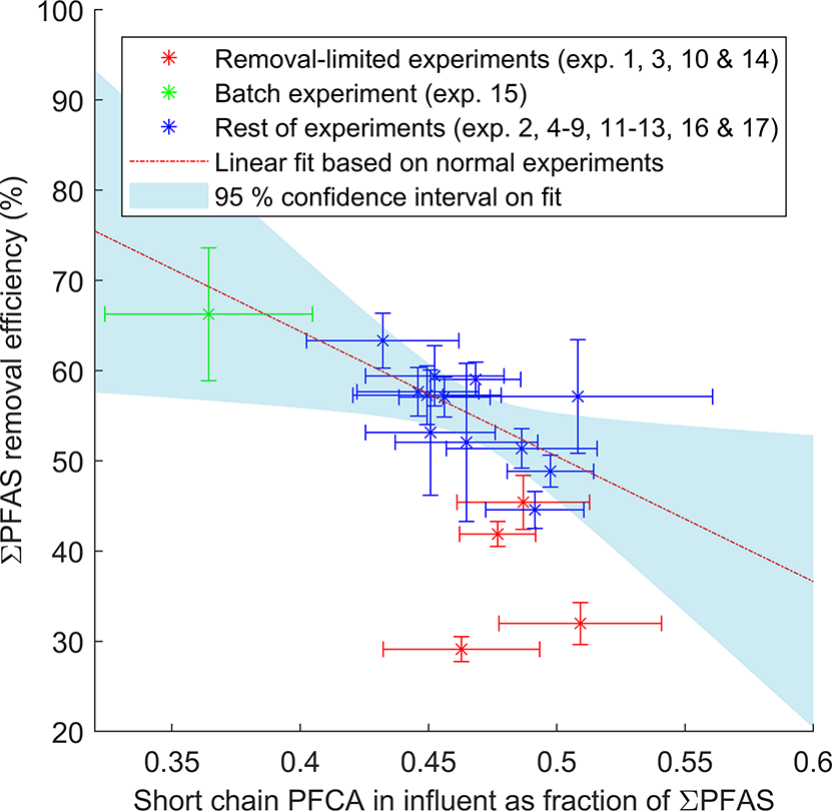
ΣPFAS removal efficiency (%) as a function of fraction short-chain PFCA in total influent PFAS. The correlation fit is only based on experiments without process limitations on the removal, represented in blue in this plot (see Table 1). Horizontal error bars represent the standard deviation of the short-chain PFCA fraction, and vertical error bars represent the standard deviation of ΣPFAS removal efficiency.

On the basis of the results presented in Figure 2, all experiments representing the lowest removal efficiencies in Figure 2A–D were deemed process-limited. Therefore, 12 continuous experiments without process-induced limitations on the RE were selected for statistical analysis (experiments 2, 4–9, 11–13, 16, and 17 in Table 1). All these 12 experiments have a *t*_c_, %_foam_, and *Q*_air_ of at least 15 min, 10%, and 7.5 L min^–1^, respectively.On the basis of these experiments, a significant negative correlation (Pearson’s *r* = −0.63 (95% CI: −0.88 to −0.09), *p* < 0.05) between the fraction short-chain PFCA in the influent ΣPFAS and the ΣPFAS RE was found, as illustrated in Figure 3. Hence, water types with a high fraction of long-chain compounds may thus be more suitable for foam fractionation treatment than the leachate water used in the current study. Even commercially available batch foam fractionation processes have a lower removal of short-chain PFAS in comparison to long-chain PFAS.^36,47^

**Figure 4:**
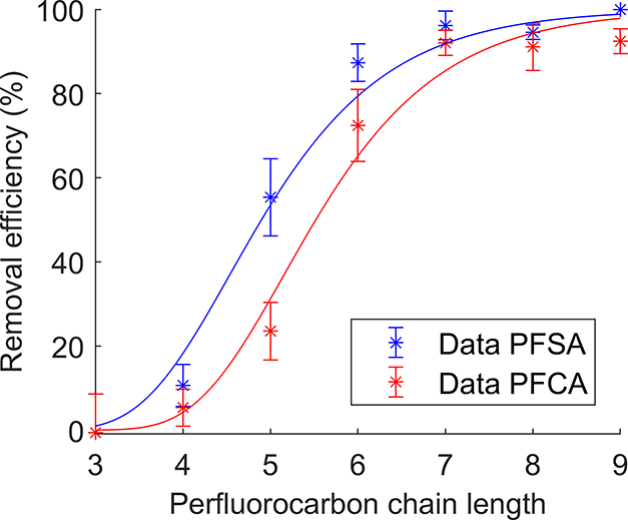
PFCA and PFSA removal as a function of perfluorocarbon chain length. Only the results from experiments without process-induced limitations were included in this plot (i.e., Exp. 2, 4–9, 11–13, 16, and 17, Table 1). The solid lines are model fits to eq 7, with the optimized parameter *a* at 9400 and 5100 for PFCA and PFSA, respectively. Error bars represent the standard deviation between experiments.

The relationship between perfluorocarbon chain length and RE is further illustrated in Figure 4. These results confirm the literature finding that PFAS removal efficiencies decrease exponentially with perfluoroalkyl chain length in foam fractionation,^3,33,40−42^ with a fit as given in eq 7. For readability, only PFCA and PFSA were included in Figure 4, but a more complete plot is given in Figure S2. Although the comparatively low influent concentrations of most non-PFAA PFAS causes high variability in some of the results, similar dependencies on perfluorocarbon chain length were found for the RE of 4:2 FTSA, 6:2 FTSA, 8:2 FTSA, FOSA, MeFOSAA, and EtFOSAA, as also shown in Figure S2.

PFOS and PFOA had average removal efficiencies of 95 ± 2% and 92 ± 3%, at mean influent concentrations of 230 ± 110 and 630 ± 150 ng L^–1^, respectively. The REs for other C_8_ PFAS (8:2 FTSA, FOSA, MeFOSAA, and EtFOSAA) were similarly high. PFNS was only detected at quantifiable concentrations in two influent and 28 foam samples but not in any effluent samples and was thus assumed to have a removal efficiency of 100%. These results correspond well with the literature findings in other foam fractionation studies of >90% removal for long-chain PFCA and PFSA but lower or no removal of short-chain compounds.^33,36,40,42^ PFCA of the same carbon number were removed to a lower extent than their PFSA equivalent, which has also been shown previously.^33,34,40,42^ This phenomenon is due to PFSA having higher adsorption coefficients to water–air interfaces because of their higher hydrophobicity.^33,34^ This effect was more pronounced for shorter chain lengths, as visible in Figure 4.

### 3.4 Enriching versus Stripping Modes

**Figure 5:**
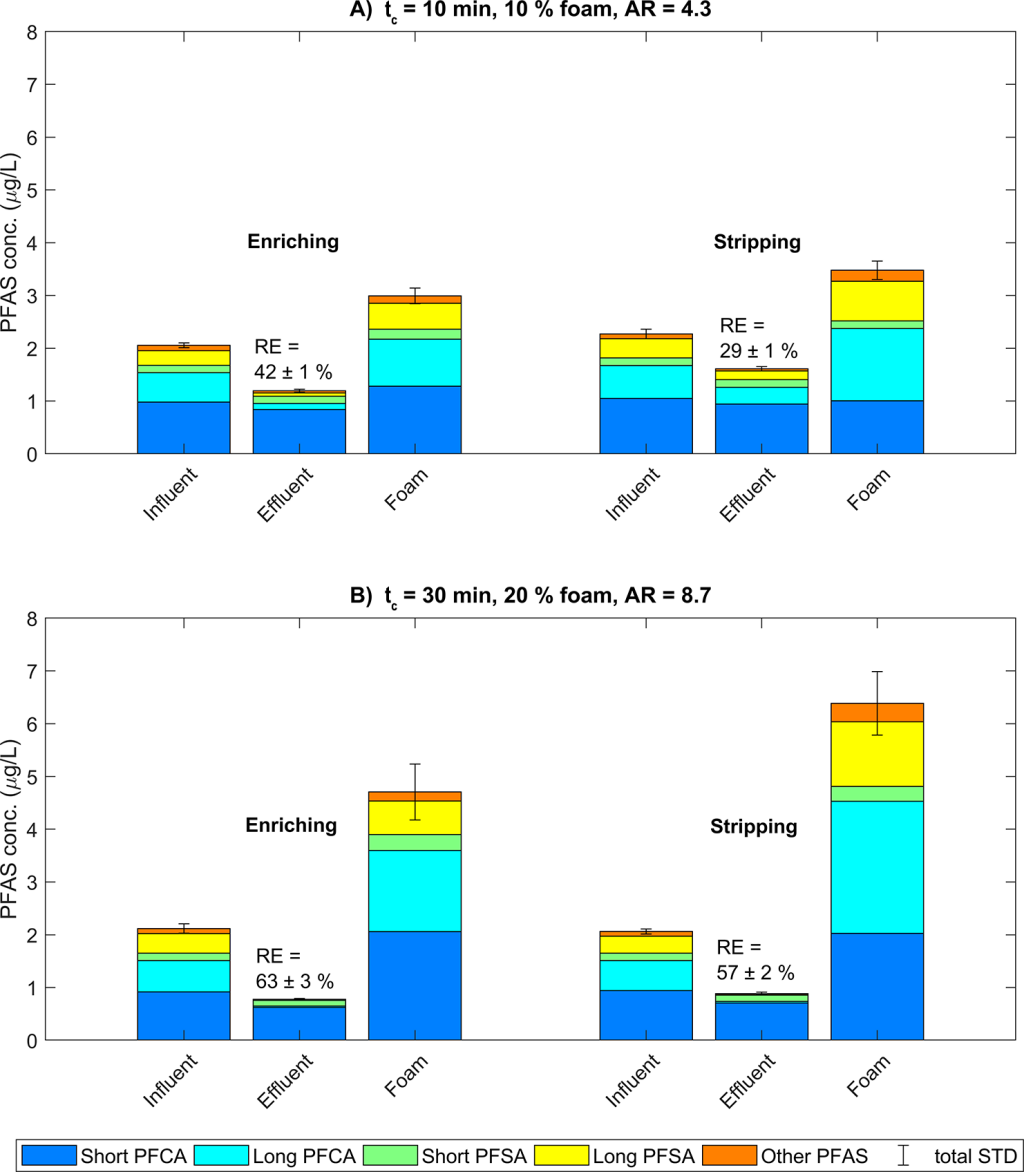
Comparison between experiments in enriching (A) Exp. 1 and (B) Exp. 5, Table 1)) and stripping (A) Exp. 3 and (B) Exp 4, Table 1)) modes under otherwise identical conditions. (A): 10 min contact time (*t*_c_), 10% foam, air ratio (AR) 4.3. (B): 30 min *t*_c_, 20% foam, AR 8.7. Error bars represent the standard deviation of the ΣPFAS concentration. For the classification of all PFAS, see SI Section F.

Two experiments were carried out in stripping mode, i.e., with the water influent above the air/water interface, as well as in enriching mode under otherwise identical conditions (experiments 3 and 4 (stripping) and 1 and 5 (enriching) in Table 1). In both comparisons, the mean ΣPFAS removal was higher in enriching mode than in stripping mode (Figure 5). These differences are not in accordance with the literature, which predicts a higher removal of contaminants in stripping mode compared to enriching mode, because the liquid between the foam bubbles has a higher PFAS concentration in stripping mode.^35,36^ In the current system, the foam layer was not sufficiently stable, so introduction of the influent above the foam surface lead to an observable collapse of the foam. Improvements of the column, such as introducing an inlet valve higher above the interface on the opposite side of the foam outlet and a foam outlet above this inlet valve, may prevent the foam from collapsing and result in improved performance in stripping mode. Moreover, introducing a vacuum pump for continuous foam collection may also increase the removal, as shown by McCleaf et al.^40^

### 3.5 Batch Mode

**Figure 6:**
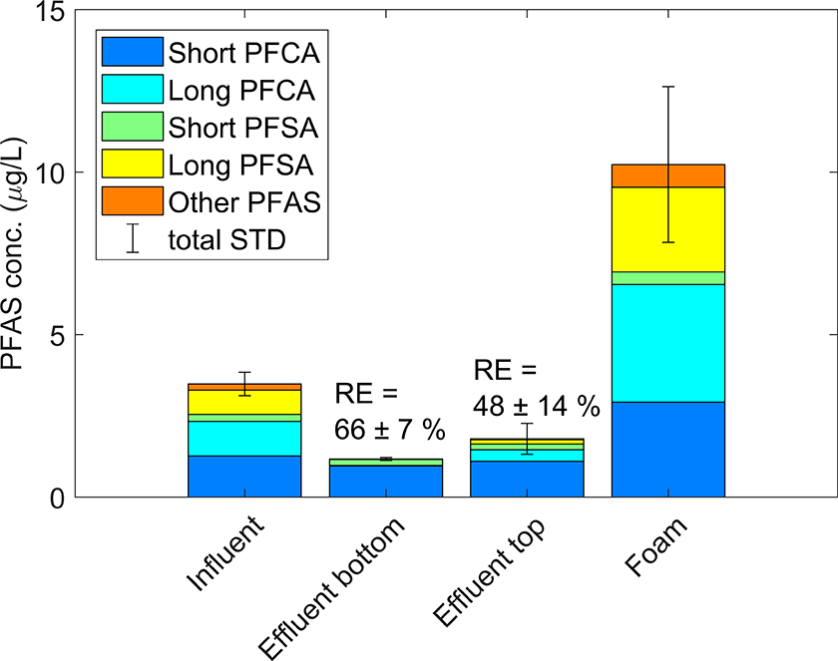
Removal efficiency in batch test (*t*_c_ 20 min, Q_air_ 20 L min^–1^, 3% foam). Error bars represent the standard deviation of the ΣPFAS concentration. For the classification of all PFAS, see SI Section F.

The ΣPFAS RE of the benchmark test in batch mode was 66 ± 7%, as shown in Figure 6. This result was not significantly different from the continuous test with the highest removal (Figure 5B, *p* > 0.05, Welch *t* test). Furthermore, as shown in Figure 3, the fraction short-chain PFAS was lowest of all experiments in the batch test, which may have increased the ΣPFAS RE. Moreover, the removal in batch mode strongly depended on where the effluent samples are taken. After turning off the air flow, effluent taken from the bottom of the column had lower PFAS concentrations than effluent taken from the top of the column, which only had a removal of 48 ± 14%. It may thus be possible to increase the batch-mode removal by collecting a higher foam fraction, but the limitation of low short-chain PFCA removal was not reduced in batch operation.

### 3.6 Mass Balance

The mass balance did not close for all experiments. The mass balance for the continuous tests ranged from 66 ± 7% to 104 ± 10%, for experiments 12 and 10 (Table 1), respectively. For the batch experiment, the mass balance only closed to 42 ± 15% when considering the bottom effluent samples or 59 ± 22% when considering the top effluent samples. The preliminary continuous experiment showed an enrichment of some PFAS in the water layer at the top of the column relative to the effluent, as shown in Figure S1. These PFAS will not show up in the mass balance, since they are neither in the foam nor in the effluent, and their accumulation may thus lead to a lower mass balance closure. It could be hypothesized that collecting higher foam fractions would thus improve the mass balance closure, but no significant effect of the %_foam_ on the mass balance was found (one-way ANOVA, *p* > 0.05), and in the batch test, the mass balance did not close either when only considering the top layer concentrations.

The overall mass balance did not correlate with ΣPFAS removal either, but individual balances closed significantly better for compounds with a lower removal, i.e., short-chain compounds (Pearson’s *r* = −0.95 (95% CI: −0.96 – −0.94), *p* < 0.05). For perfluorocarbon chain lengths up to 10 for PFCA and up to 8 for PFSA, one-way ANOVA showed a statistically significant difference in mass balance closure with increasing chain length (F(7,88) = 14 for PFCA, F(4,55) = 21 for PFSA, *p* < 0.05 for both). PFAS with perfluorocarbon chain lengths above the specified numbers were excluded because of their relatively low influent concentrations of <5 ng L^–1^, and only the results from experiments without process-induced limitations (Exp. 2, 4–9, 11–13, 16, and 17, Table 1) were included in these calculations. Box plots and details on the statistical calculations are given in SI Section I.

Since long-chain PFAS were removed better and were thus more enriched at the air–water surface, these strong correlations indicate that PFAS accumulating at the air–water interface may also escape to the air as aerosols rather than being captured in the foam. This hypothesis is supported by the work of Ebersbach et al.,^42^ who demonstrated the aerosol-mediated removal of 6:2 FTSA, PFOS, and PFOA from concentrated water. Moreover, aerosol enrichment with PFAS is a well-documented phenomenon, both in nature and in engineered systems.^48−50^ The presence of aerosols in the current system was visible from the bursting of foam bubbles, leading to the formation of droplets and bubbles on the lid and upper walls of the column. However, McCleaf et al.^40^ found no significant PFAS concentrations in their aerosol trap after foam fractionation, which may be related to their use of a vacuum pump for foam collection. In addition to loss of PFAS in aerosols, the complex water matrix may have caused transformation of certain compounds as a result of oxidation, which could have further skewed the mass balance.

## 4 Conclusions

This study set out to examine the applicability of pilot-scale continuous foam fractionation for treatment of PFAS-contaminated leachate water. It was shown that treatment efficiency decreased with decreasing contact time, air flow rate and collected foam fraction. Long-chain compounds were removed better than short-chain PFAS, and PFSA were removed more efficiently than PFCA. PFOS and PFOA had average removal efficiencies of 95% and 92%, but no removal of PFBA and only 10% removal of PFBS were found. No improvement in treatment efficiency was found when operating in batch mode, which indicates that continuous operation is a viable alternative for commercially available batch systems. Despite the relatively low ΣPFAS removal of approximately 60%, the results indicate a high applicability of continuous foam fraction, especially for treatment of water types contaminated with mainly long-chain PFAS. Further research is required to confirm if the high long-chain PFAS removal extends to water types with different water quality matrixes and PFAS concentrations than the investigated leachate.

Currently, most regulations for aqueous PFAS emissions to the environment still include almost exclusively long-chain PFAS. For example, the European Water Framework Directive defined an average annual PFOS concentration in inland surface water of 0.65 ng L^–1^ as the environmental quality limit.^51^ In The Netherlands, soil-washing facilities are allowed to discharge 4000 m^3^ of wastewater containing at most 500 ng L^–1^ PFOS, 500 ng L^–1^ PFOA, and 1000 ng L^–1^ HFPO–DA (GenX) annually.^52^ In the United States, efforts are underway to enforce remediation of PFOS and PFOA releases into the environment.^16^ In the most effective continuous experiment presented in this study, mean PFOS and PFOA concentrations decreased from 230 and 580 to 7 and 20 ng L^–1^, respectively, which falls well within the Dutch standards for soil washing wastewater. However, landfill facilities often have specific individual discharge permits for PFAS, so drawing generalized conclusions on the treatment performance with respect to regulatory limits is difficult.

The greatest advantage of the presented technology is its simplicity. Aeration is common in most wastewater treatment facilities, and for plants, treating PFAS contaminated water introducing a foam fractionation process is thus an easily implemented and economical way to decrease PFAS emissions to the environment. Possibly, this technology can even be integrated with aeration steps that are already applied on-site, by installing an appropriate foam collection system. Naturally, the collected foam would need further treatment, where the reduced volume of approximately 10% of the total inlet volume allows relatively smaller on-site degradative treatment of the coalesced foam, as exemplified in previous studies.^32,33^

Two of the most promising degradative treatment technologies for PFAS-contaminated water are plasma treatment and electrochemical oxidation.^53^ Both these technologies have been applied successfully to leachate water matrices similar to the foam produced in this study, with ΣPFAS concentrations in the low μg L^–1^ range, albeit at higher TOC concentrations and conductivities.^54,55^ Both these destructive technologies were more effective for the removal of long-chain as compared to short-chain PFAS. A drawback of electrochemical degradation was the formation of short-chain compounds as degradation products, which was not observed in plasma treatment. These results indicate that degradative treatment of the foam produced, as described here, will most probably be possible.

Further research should focus on improving the removal of short-chain compounds in the foam fractionation process. Alternative methods for the introduction of air bubbles, such as electrochemical bubble formation, may lead to higher available surface area and thus higher removal. Possibly, this will increase the removal of short-chain compounds. The use of image processing technologies for determining the size distributions in bubbly flows could enhance the understanding of the effect of bubble size on removal.^56,57^ Alternatively, additives such as metal activators may be tested, which have been shown to increase the removal of long-chain compounds.^40,43^ Enhanced foam collection, for example with a vacuum pump, may also improve the removal of short-chain PFAS, as may combining several foam fractionation steps in a row.

Another area for future work would be the variation in mass balance closure that was found. Introducing air as well as aerosol sampling and analyzing the PFAS concentrations in the exhaust air may be beneficial toward closing the mass balance and could indicate if any PFAS escape the system. Finally, testing other water matrixes with a higher fraction of long-chain compounds could confirm the presented limitation of low short-chain removal.

## Supporting Information Available

Figures of overview of results of preliminary experiment, PFAS removal as a function of chain length, and boxplots for the different individual PFAS included in the ANOVA analysis for the effect of chain length on mass balance, tables of dates of experiments, scheduled multiple reaction monitoring transitions for LC–MS/MS analysis of PFAS concentrations, internal standards used for compounds without a corresponding mass labeled internal standard in the IS mixture, overview of instrument parameters for quantification of PFAS, general chemistry results of analyzed samples, legitimization of sample exclusion, overview of PFAS designation into groups, and fitted parameters for the effect of process variables on ΣPFAS removal, and discussion of analytical method (PDF)
