# Supplementary material for: Pilot-Scale Continuous Foam Fractionation for the Removal of Per- and Polyfluoroalkyl Substances (PFAS) from Landfill Leachate
Source: ACS ES T Water. 2022 May 4;2(5):841–51. doi: 10.1021/acsestwater.2c00032 (PMC9112282; doi:10.1021/acsestwater.2c00032)
Supplement: Supplementary file 1 — ew2c00032_si_001.pdf [file ew2c00032_si_001.pdf]

Supplementary Information to *Pilot-scale continuous foam fractionation for the removal of per- and polyfluoroalkyl substances (PFAS) from landfill leachate*

Sanne Smith[1], Karin Wiberg[1], Philip McCleaf[2]and Lutz Ahrens[1]

[1]Department of Aquatic Sciences and Assessment, Swedish University of Agricultural Sciences (SLU), P.O. Box 7050, SE-750 07, Uppsala, Sweden
[2]Uppsala Water and Waste AB, P.O. Box 1444, SE-751 44, Uppsala, Sweden
E-mail contact: sanne.smith@slu.se

A. Results preliminary experiment

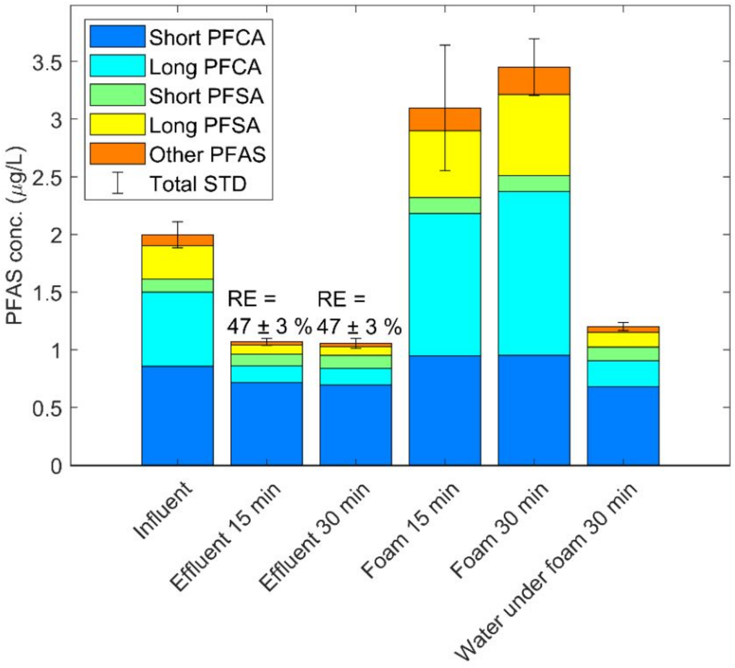

Figure SI. 1: Overview of results of preliminary experiment (t_c = 10 min, 30 % foam, AR 2.16, Q_{air} 10 L min^{-1}). Error bars represent the standard deviation on the ΣPFAS concentration.

Figure SI. 1 summarizes the results of the preliminary triplicate experiments at 10 minutes contact time (t_c), air flow 10 L min^{-1} (Q_{air}) and 30 % foam. For details on the experimental approach, see main text section 2.2. PFHxA, PFOA, PFDA, PFPeS, PFHxS, PFHpS, PFOS, PFECHS, FOSA, MeFOSAA and EtFOSAA concentrations were significantly higher directly under the air/water surface than in the effluent samples for at least one of the time points. However, since the effluent quality remained constant, this was deemed not to affect treatment performance in continuous mode. Moreover, ΣPFAS removal directly

under the air/water surface was 40 ± 2.5 %, indicating that removal already takes place in the top part of the column. Hence, further tests were carried out as described in main text section 2.2, with replicate samples taken at four different time points instead of in experimental triplicates.

B. Overview of experiments

Table SI. 1: Dates of experiments. Experiments that were deemed process-limited are indicated with an asterisk.

Exp.	Date
**0**	2021-04-12
**1***	2022-03-11
**2**	2021-08-24
**3***	2021-08-24
**4**	2021-08-30
**5**	2021-08-30
**6**	2021-08-30
**7**	2021-09-09
**8**	2021-09-09
**9**	2021-09-09
**10***	2021-09-14
**11**	2021-09-14
**12**	2021-09-14
**13**	2021-09-27
**14***	2021-09-27
**15**	2021-09-27
**16**	2021-10-13
**17**	2021-10-13

C. Analytical Method

Water samples were sonicated for 5 min before filtration. After filtration, 100 µL of the IS mixture (main text Section 2.2) at 50 ng mL^{-1} concentration for individual IS in methanol was added to each sample, the samples were then vigorously mixed and sonicated again for 5 min. Oasis® WAX cartridges (6 mL, 150 mg, 30 µm, Waters) were preconditioned with 4 mL 0.1 % ammonium hydroxide in methanol, followed by 4 mL methanol and 4 mL Milli-Q water. After sample loading at approximately one drop per second, the cartridges were washed with 4 mL 25 mM ammonium acetate buffer in Milli-Q water. The cartridges were dried under vacuum and eluted with 4 mL methanol and 4 mL 0.1% ammonium hydroxide in methanol. The extracts were concentrated to 1 mL volume under nitrogen. A lab blank of 150 mL Milli-Q water was included in each run.

The limit of quantification (LOQ) was 0.33 ng L^{-1} for the foam samples (150 mL analyzed) and 0.2 ng L^{-1} for the influent and effluent samples (250 mL analyzed). The LOQ was set to the lowest concentration of the calibration curve with a consistent signal-to-noise ratio of 10. As explained in the main text, concentrations below the LOQ were taken as zero, which introduces an error. The lowest ΣPFAS concentration found in any of the samples was 750 ng L^{-1}, with 15 of the PFAS in the method being non-detects. The maximum concentration of the non-detects in this effluent sample thus contributed 0.4 % to

the total PFAS concentration, which was deemed negligible; hence non-detect concentrations were set to zero.

120 mL Milli-Q samples spiked with 6.25, 12.5 and 25 ng of each native PFAS included in the analysis were extracted and analyzed for their recovery. For all compounds, the mean and median absolute deviation from the spiked concentrations were 9.0 % and 5.7 %, respectively. The only compounds with mean absolute deviations higher than 15 % were HFPO-DA, PFECHS, PFDS, 11Cl-PF3OUdS and PFTriDA, of which only PFDS and PFTriDA were above 20 % (-26 % and 39 % mean deviation, respectively). The maximum contribution of these compounds to the ΣPFAS concentrations in all samples were only 0.004 % and 3 %, respectively, but nonetheless, the concentrations of these compounds should be considered to have a lower degree of accuracy.

Table SI. 2: Scheduled multiple reaction monitoring transitions for LC-MS/MS analysis of PFAS concentrations. The compounds are ordered on precursor mass.

Compound	Precursor mass (Q1, m/z)	Product mass (Q3, m/z)	Retention time (min)	Declustering potential (V)	Collision energy (V)	Collision cell exit potential (V)
**Perfluorobutanoic acid (PFBA)**	213.0	168.9	1.06	-20	-12	-5
**$^{13}C_4$-PFBA**	217.0	172.0	1.06	-25	-14	-9
**Perfluoropentanoic acid (PFPeA)**	262.9	218.9	1.27	-5	-12	-11
**$^{13}C_5$-PFPeA**	268.0	223.0	1.27	-30	-12	-9
**Perfluorobutane sulfonate (PFBS)**	299.0	80.0	1.30	-90	-64	-7
**PFBS**	299.0	99.0	1.30	-90	-50	-9
**$^{13}C_3$-PFBS**	302.0	80.0	1.30	-90	-70	-5
**Perfluorohexanoic acid (PFHxA)**	313.0	268.9	1.56	-35	-10	-14
**PFHxA**	313.0	118.9	1.56	-35	-26	-9
**$^{13}C_5$-PFHxA**	318.0	273.0	1.56	-35	-14	-13
**4:2 Fluorotelomer sulfonic acid (4:2 FTSA)**	327.0	307.0	1.53	-75	-28	-11
**4:2 FTSA**	327.0	287.0	1.53	-75	-28	-11
**hexafluoropropylene oxide dimer acid (HFPO-DA)**	328.9	284.9	1.66	-20	-8	-13
**HFPO-DA**	328.9	169.0	1.66	-20	-8	-13
**$^{13}C_2$-4:2 FTSA**	329.0	309.0	1.53	-5	-28	-13
**$^{13}C_3$-HFPO-DA**	332.0	287.0	1.66	-15	-8	-9
**Perfluoropentane sulfonate (PFPeS)**	349.0	80.0	1.57	-100	-76	-7
**PFPeS**	349.0	99.0	1.57	-100	-66	-7
**Perfluoroheptanoic acid (PFHpA)**	362.9	318.9	1.93	-40	-14	-11

**PFHpA**	362.9	169.0	1.93	-40	-24	-5
**$^{13}C_4$-PFHpA**	367.0	172.0	1.93	-40	-24	-7
**4,8-dioxa-3H-perfluorononanoic acid (NaDONA)**	376.9	250.9	1.96	-45	-18	-9
**NaDONA**	376.9	84.9	1.96	-45	-54	-7
**Perfluorohexane sulfonate (PFHxS)**	399.0	79.9	1.92	-95	-82	-5
**PFHxS**	399.0	99.0	1.92	-115	-70	-7
**$^{13}C_3$-PFHxS**	402.0	80.0	1.92	-105	-86	-7
**Perfluorooctanoic acid (PFOA)**	413.0	369.1	2.32	-45	-16	-15
**PFOA**	413.0	169.1	2.32	-40	-24	-5
**$^{13}C_8$-PFOA**	421.0	376.0	2.32	-45	-16	-17
**6:2 Fluorotelomer sulfonate (6:2 FTSA)**	427.0	81.0	2.28	-85	-72	-7
**$^{13}C_2$-6:2 FTSA**	429.0	81.0	2.28	-90	-70	-7
**Perfluoroheptane sulfonate (PFHpS)**	448.9	80.0	2.32	-130	-90	-7
**PFHpS**	448.9	99.0	2.32	-125	-80	-7
**Perfluoroethyl-cyclohexane sulfonate (PFECHS)**	461.0	381.0	2.26	-90	-38	-13
**PFECHS**	461.0	99.0	2.26	-95	-68	-7
**Perfluorononanoic acid (PFNA)**	463.0	219.0	2.71	-50	-24	-9
**PFNA**	463.0	168.9	2.71	-45	-26	-9
**$^{13}C_9$-PFNA**	472.0	172.0	2.71	-50	-26	-9
**Perfluorooctane sulfonamide (FOSA)**	498.0	78.0	2.91	-105	-86	-7
**Perfluorooctane sulfonate (PFOS)**	499.2	80.0	2.71	-130	-110	-7
**PFOS**	499.2	98.9	2.71	-115	-94	-9
**$^{13}C_8$-FOSA**	506.0	77.9	2.91	-110	-82	-7
**$^{13}C_8$-PFOS**	507.0	80.0	2.71	-135	-108	-7
**Perfluorodecanoic acid (PFDA)**	513.0	268.9	3.09	-55	-26	-11
**PFDA**	513.0	218.9	3.09	-50	-26	-9
**$^{13}C_6$-PFDA**	519.0	474.0	3.09	-75	-14	-19
**8:2 Fluorotelomer sulfonate (8:2 FTSA)**	527.2	506.9	3.08	-125	-38	-15
**8:2 FTSA**	527.2	81.0	3.08	-110	-88	-7
**$^{13}C_2$-8:2 FTSA**	529.0	81.0	3.08	-120	-86	-7
**9-chloro-hexadecafluoro-3-oxanonane sulfonate**	531.0	351.0	2.92	-110	-36	-13

**(9Cl-PF3ONS)**						
**9Cl-PF3ONS**	531.0	83.0	2.92	-105	-78	-7
**Perfluorononane sulfonate (PFNS)**	549.0	80.0	3.07	-140	-110	-7
**PFNS**	549.0	99.0	3.07	-135	-92	-7
**Perfluoroundecanoic acid (PFUnDA)**	563.0	519.0	3.43	-60	-18	-9
**PFUnDA**	563.0	268.9	3.43	-55	-26	-11
**N-methyl-perfluorooctane sulfonamido acetic acid (MeFOSAA)**	569.9	418.9	3.26	-90	-28	-21
**MeFOSAA**	569.9	482.9	3.26	-90	-22	-21
**$^{13}C_7$-PFUnDA**	570.0	525.0	3.43	-90	-18	-9
**D_3-MeFOSAA**	573.0	419.0	3.26	-80	-28	-19
**N-ethyl-perfluorooctane sulfonamido acetic acid (EtFOSAA)**	584.0	419.0	3.44	-85	-28	-17
**EtFOSAA**	584.0	219.0	3.44	-85	-36	-9
**D_5-EtFOSAA**	589.0	419.0	3.44	-85	-30	-15
**Perfluorodecane sulfonate (PFDS)**	598.9	80.0	3.41	-150	-120	-7
**PFDS**	598.9	99.0	3.41	-155	-114	-7
**Perfluorododecanoic acid (PFDoDA)**	613.0	569.0	3.74	-60	-18	-11
**PFDoDA**	613.0	318.9	3.74	-65	-28	-13
**$^{13}C_3$-PFDoDA**	615.0	570.0	3.74	-65	-18	-11
**11-chloro-eicosafluoro-3-oxaundecane-1-sulfonic acid (11Cl-PF3OUdS)**	630.9	450.9	3.59	-115	-42	-19
**11Cl-PF3OUdS**	630.9	83.0	3.59	-120	-80	-7
**Perfluorotridecanoic acid (PFTriDA)**	662.9	618.9	4.03	-70	-18	-11
**PFTriDA**	662.9	319.0	4.03	-70	-32	-11
**Perfluorotetradecanoic acid (PFTeDA)**	713.0	668.9	4.29	-75	-20	-9
**PFTeDA**	713.0	368.8	4.29	-75	-30	-13
**$^{13}C_2$-PFTeDA**	715.0	670.0	4.29	-75	-20	-13

Table SI. 3: Internal standards used for compounds without a corresponding mass labeled internal standard in the IS mixture.

Compound	IS used
PFPeS	$^{13}C_3$-PFHxS
NaDONA	$^{13}C_3$-HFPO-DA
PFHpS	$^{13}C_8$-PFOS
PFECHS	$^{13}C_8$-PFOS
9Cl-PF3ONS	IS_FOSA
PFNS	$^{13}C_8$-PFOS
PFDS	$^{13}C_8$-PFOS
11Cl-PF3OUdS	$^{13}C_8$-FOSA
PFtriDA	$^{13}C_2$-PFTeDA

Table SI. 4: Overview of instrument parameters for quantification of PFAS

**Instrument**	Sciex Triple Quad™ 3500 LC-MS/MS (USA)		
**Guard column**	Phenomenex KJ0-4282		
**Precolumn**	Phenomenex Kinetix® 1.7 µm C18 100 Å		
**Analytical column**	Phenomenex Gemini® 3 µm C18 110 Å		
**Autosampler temperature**	15 °C		
**Injection volume**	10 µL		
**Flow rate**	0.6 mL min^{-1}		
**Column oven temperature**	40 °C		
**Mobile phase**	A: 10 mM ammonium acetate in MilliQ water; B: 100 % methanol		
**Gradient program**	Time (min)	%A	%B
	0	95	5
	0.1	45	55
	4.5	1	99
	8	1	99
	8.5	95	5
	9	95	5
**Ionization**	Heated electrospray ionization in negative mode		
**Negative ion spray voltage**	-3000 V		
**Curtain gas pressure**	35 psi		
**Collision gas pressure**	8 psi		
**Gas temperature**	600 °C		
**Ion source gas 1 pressure**	30 psi		
**Ion source gas 2 pressure**	40 psi		
**Run time**	9 min		
**Calibration curve concentrations**	0.01, 0.05, 0.1, 0.5, 1, 5, 10, 50 & 100 ng mL^{-1}		

D. General Chemistry

Table SI. 5: General chemistry results of analyzed samples

	Influent				Effluent				Foam	
**Experiment**	**0**	**1**	**13**	**15**	**0**	**1**	**13**	**15**	**13**	**15**
**DOC (mg L^{-1})**	36	35	37	36	36	33	38	39	42	49
**Phosphor (µg L^{-1})**	200	117	132	96	200	76	104	104	173	202
**Calcium (mg L^{-1}**	156	150	154	149	157	137	155	153	154	151
**Manganese (µg L^{-1})**	442	487	636	526	432	351	646	681	633	586
**Sodium (mg L^{-1})**	640	702	743	759	647	697	751	754	730	746
**Potassium (mg L^{-1})**	222	222	251	264	224	220	276	258	266	257
**Iron (mg L^{-1})**	5.1	5.8	5.3	5.2	4.5	3.6	5.9	5.4	9.2	10
**Aluminum (µg L^{-1})**	63	14	21	9.4	59	14	16	10	26	61
**Copper (µg L^{-1})**	26	13	15	161	28	15	53	16	76	78
**Magnesium (mg L^{-1})**	50	57	59	60	50	57	61	60	59	60
**Hardness (°dH)**	33	34	35	35	34	32	36	35	35	35
**NO_2 (mg L^{-1})**	0.65	0.37	0.76	0.68	0.76	0.39	0.68	0.66	0.69	0.71
**COD-Mn (mg L^{-1})**	38	19	25	27	46	19	28	25	31	31
**Ammonium (mg L^{-1})**	62	56	58	60	62	55	61	61	61	60
**Phosphate (mg L^{-1})**	0.15	0.06	<0.04	<0.04	0.18	0.06	<0.04	<0.04	<0.04	<0.04
**Nitrate (mg L^{-1})**	16	21	17	20	17	21	17	17	17	17
**Fluoride (mg L^{-1})**	<0.5	<0.5	<0.5	<0.5	<0.5	<0.5	<0.5	<0.5	<0.5	<0.5
**Chloride (mg L^{-1})**	818	909	909	1050	834	915	950	950	950	948
**Sulphate (mg L^{-1})**	202	111	121	99	199	112	93	89	98	92
**Turbidity (FNU)**	56	27	32	24	41	37	59	51	55	106
**Conductivity (mS m^{-1})**	403	484	430	436	425	477	446	450	441	443
**pH**	7.7	8.0	7.7	8.0	8.0	8.1	8.0	7.7	8.0	8.0
**Alkalinity (mg L^{-1})**	1280	1330	1370	1260	1260	1230	1380	1480	1410	1390
**TOC (mg L^{-1})**	26	45	38	37	26	33	39	39	43	52
**Uranium (µg L^{-1})**	26	43	30	29	26	43	30	30	29	30

E. Overview of excluded data

Table SI. 6: Legitimization of sample exclusion. In addition to these samples, some foam extracts had too high concentrations of PFOA (>> 100 ng mL^{-1}), which made quantification with a quadratic calibration curve impossible. For these samples, the PFOA calibration curve was changed to linear. Unrealistically high values indicate a value that is at least a factor 5 or 400 ng L^{-1} higher than the mean concentration of the remaining three samples of the same type.

Experiment number	Excluded sample	Reason of exclusion
**4**	Foam 2 hr	Lost during collection due to human error
**10**	Influent 2 hr	Lost during analysis due to a production error in the polypropylene tube used for collection after extraction
	Effluent 0.5 hr	Contaminated in lab – unrealistically high PFNA, FOSA, PFOS, PFDA, PFUnDA and PFDoDA concentrations
	PFBA in effluent 1 hr	Unrealistically high value - replaced by concentration of 0.5 hr sample (which was otherwise excluded)
	PFBA in foam 2 hr	Unrealistically high value - excluded
**11**	Effluent 2 hr	Contaminated in lab – unrealistically high PFOA, PFNA, FOSA, PFOS, PFDA, PFUnDA and PFDoDA concentrations
	PFTeDA in effluent 1 hr	Unrealistically high value – replaced by concentration of 2 hr sample (which was otherwise excluded)
**15**	Bottom effluent r2	Not spiked with IS prior to extraction

In total, 204 PFAS samples were analyzed for the study, of which five have been excluded completely and three have one excluded compound.

F. Classification of PFAS

Table SI. 7: Overview of PFAS designation into groups

Group	Compounds
**Short carboxylates**	PFBA, PFPeA, PFHxA, PFHpA
**Long carboxylates**	PFOA, PFNA, PFUnDA, PFDoDA, PFTriDA, PFTeDA
**Short sulfonates**	PFBS, PFPeS
**Long sulfonates**	PFHxS, PFHpS, PFOS, PFNS, PFDS
**Rest**	4:2 FTSA, 6:2 FTSA, 8:2 FTSA, FOSA, HFPO-DA, NaDONA, PFECHS, 9Cl-PF3ONS, 11ClPF3OUdS, Me-FOSAA, Et-FOSAA

G. Fitted parameters

Table SI. 8: Fitted parameters for the effect of process variables on ΣPFAS removal, Equation 6 in the main text.

Process variable	k	RE_{Max} (%)
**Contact time at constant AR**	0.12 min^{-1}	60.5
**Contact time at constant AF**	0.11 min^{-1}	60.9

**Foam fraction**	0.33	57.1
**Air flow**	0.23 min L^{-1}	56.7

H. PFAS removal as a function of chain length

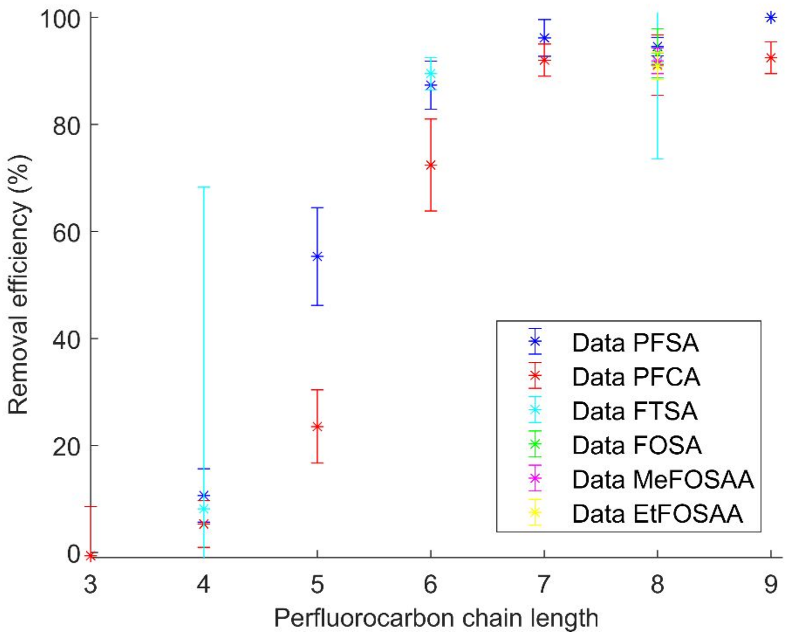

Figure SI. 2: PFAS removal as a function of chain length. Note that PFNA, PFOS, 8-2 FTSA, FOSA, MeFOSAA and EtFOSAA all have a perfluorocarbon chain length of 8. The mean influent concentration of 4-2 FTSA was only 0.8 ng L^{-1}, which caused a high standard deviation in the results as compared to compounds with higher initial concentrations.

I. Mass balance for individual compounds

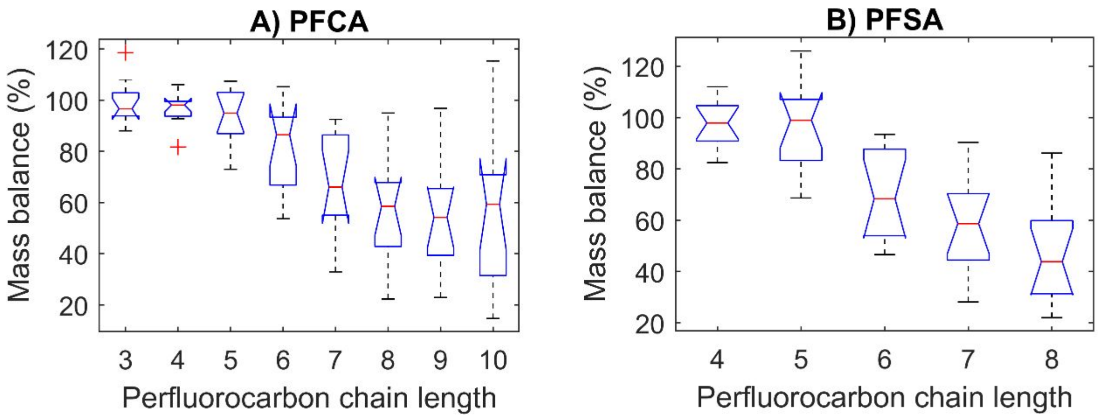

Figure SI. 3: Boxplots for the different individual PFAS included in the Anova analysis for the effect of chain length on mass balance. Note that the red lines represent medians, whereas the statistical analysis is based on mean values. The groups include the mean individual mass balances from experiments 2, 4-9, 11-13, 16 and 17, e.g. n = 12 for each group.

Based on the Anova results outlined in the main text, a statistically significant difference was found with at least 3 other groups for each PFCA group and at least 2 other groups for each PFSA group (Tukey's honestly significant difference procedure).
